# *Pichia kudriavzevii* (JD2) Immobilized on Acid Activated Perlite as a Biosorbent for Solid Phase Extraction of Cr(III) Determination by AAS (Atomic Adsorption Spectroscopy)

**DOI:** 10.1002/jemt.24815

**Published:** 2025-02-11

**Authors:** Aliya Amanzhol, Özcan Yalçınkaya, Berat Çinar Acar, Zehranur Yuksekdag

**Affiliations:** ^1^ Graduate School of Natural and Applied Science, Department of Chemistry Gazi University Ankara Türkiye; ^2^ Department of Chemistry, Science and Art Faculty Gazi University Ankara Türkiye; ^3^ Department of Biology, Science and Art Faculty Gazi University Ankara Türkiye

**Keywords:** acid treatment, adsorption, central composite design, perlite, *Pichia kudriavzevii* JD2, solid phase extraction (SPE)

## Abstract

In this study, the preconcentration of Cr(III) ion in *Pichia kudriavzevii* JD2 immobilized perlite adsorbent by solid phase extraction was investigated. The determination of Cr(III) was performed using flame atomic absorption spectrometry (AAS). The effects of pH, adsorbent amount, recovery solution volume and type, and sample solution flow rate and volume on the recovery efficiency of Cr(III) ions were investigated. Optimized preconcentration conditions for Cr(III) were established using a column technique. The optimal parameters were determined as pH 4, a recovery solution of 2 mol/L HNO_3_ with a volume of 10 mL, and a sample flow rate of 1–3 mL/min, preconcentration factor 25. Under these conditions, the recovery efficiency of Cr(III) ion on perlite immobilized with *Pichia kudriavzevii* JD2 was found to be 100.1% ± 0.3% with a 95% confidence level. Analytical variables with a limit of detection (LOD) of 4.8 μg/L and a limit of quantification (LOQ) of 15.8 μg/L were determined for the Cr(III) ion. The accuracy of the method was determined using standard reference materials (SPS‐WW1). The relative error of the recovery efficiency was determined to be less than 10%. The method was applied to the determination of Cr(III) in various water samples, such as tap water and mineral waters.

## Graphical abstract

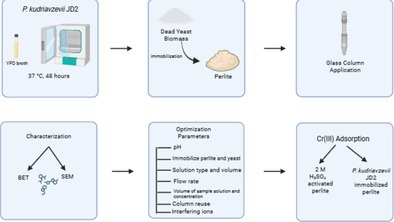

**Summary**

- Acid activation experiments were done without causing any deterioration in the crystal structure of the perlites.
- An environmentally friendly and cost‐effective adsorbent was obtained for the efficient removal of Cr(III) ions.
- The perlite immobilized with *Pichia kudriavzevii* JD2 was found to be highly efficient in recovering Cr(III) ions, achieving recovery rate of 100.1% with a 95% confidence level.

## 1 Introduction

Inorganic substances find their way into natural waters through various sources. The most significant natural contributor is the weathering process of rocks and soils directly exposed to surface waters. Additionally, atmospheric fallout, originating from both natural and human‐induced activities such as fossil fuel combustion and industrial processes, introduces substantial amounts of inorganic compounds into aquatic environments (Babuji et al. 2023). Human activities also play a major role in contaminating water bodies. The discharge of treated and untreated wastewaters, as well as disturbances caused by construction, mining, and forestry, release large quantities of inorganic pollutants into water systems. Even the breakdown of plant and animal matter contributes to the inorganic composition of these waters, albeit in smaller amounts (Zhang et al. 2023).

Natural water bodies are intricate electrolyte solutions interacting with diverse inorganic and organic matter. Inorganic substances introduced into these systems undergo complex abiotic and biological processes (Xie 2024). Heavy metals are associated with pollution and toxicity. Heavy metallic species can be divided into three types: toxic metals (such as Cr, Pb, Zn, Ni, Cd, Sn, etc.), precious metals (such as Pd, Ag, Au, etc.), and radionuclides (such as U, Th, Ra) (Muhammad et al. 2024). The detrimental impact of inorganic and organic pollutants, released from anthropogenic activities like mining, petrochemical plants, metallurgical, paper, tanning, and oil refining industries, as well as coal‐fired power stations, on both living organisms and human health is well‐established (Qasem, Mohammed, and Lawal 2021), agricultural residues, industrial discharges, urban runoff, treated and untreated household wastes, and livestock farming (Priyadarshanee and Das 2021; Tan et al. 2022).

Many metals are essential for plants; they activate the enzyme reaction and provide cation conductivity. Metals are widespread in nature and essential for both human health and modern society. Elements like iron, magnesium, and calcium are vital for human well‐being. However, heavy metals such as arsenic, cadmium, lead, mercury, chromium, and copper can be toxic in high concentrations, leading to a range of health problems including diarrhea, nausea, asthma, kidney damage, various cancers, and even death (Nik‐Abdul‐Ghani, Jami, and Alam 2021). Chromium is never found in the free state in nature. The primary mineral source of chromium is chromite, with the chemical formula (MgFe)O(Cr, Al, Fe)_2_O_3_. While widely distributed in the Earth's crust, chromium can exist in oxidation states ranging from −2 to +6, with trivalent Cr(III) and hexavalent Cr(VI) forms being the most prevalent in the environment (Singh et al. 2022; Cinar Acar and Yuksekdag 2023a). Cr(III) is relatively stable in water, while Cr(II) is highly unstable and rapidly oxidizes to Cr(III) under aerobic conditions. Extensive research has linked occupational exposure to Cr(VI) compounds with increased lung cancer mortality. While the exact carcinogenic properties of different chromium compounds and their solubility remain unclear, it is evident that exposure to a mixture of Cr(VI) compounds with varying solubilities poses the greatest risk to human health. (Govind et al. 2020; Cinar Acar and Yuksekdag 2023b).

In heavy metal enrichment can be done by several methods such as: ion exchange extraction, liquid–liquid extraction, chemical precipitation, adsorption, solid phase extraction (SPE), coagulation, and flocculation. Extraction methods are environmentally friendly, simple, and fast (Ahmad, Bhat, and Buang 2018). SPE can be accomplished for preconcentration and extraction of heavy metals from various water samples (Jagirani and Soylak 2020). A variety of innovative materials can be utilized as adsorbents, including magnetic substances and biosorbents derived from bacteria, yeast, and microbial biomass. These biosorbents are often immobilized on substrates like clay, XAD‐4 (Kocaoba 2022), pumice stone (Shoroog and Hasan 2021), and perlite (Aghabeyk, Azadmehr, and Hezarkhani 2022) to enhance their adsorption capabilities. The process of heavy metal accumulation using biosorbents is referred to as biosorption, characterized by the rapid and reversible binding of heavy metals from different water sources to the biomass surface (Morales‐Barrera, Flores‐Ortiz, and Cristiani‐Urbina 2020; Arici et al. 2020).

Organisms such as yeast, bacteria, fungi, and algae have the ability to absorb heavy and toxic metals from their surrounding water (Savastru et al. 2019). Dead biomass derived from these organisms is commonly used as a biosorbent due to its ease of storage and potential for metal ion reuse. However, nonliving biomass suffers from drawbacks including mass loss, reduced preconcentration efficiency, and handling difficulties in batch and continuous processes due to small particle size. To address these issues, immobilization techniques can be employed. By immobilizing yeast on perlite, for instance, the same solid phase can be reused multiple times in glass columns for various applications. In recent studies, the consideration of yeast as a biosorbent for the removal of heavy metals become the focus (Ningqin et al. 2020; Razieh et al. 2022). Yeast has emerged as a promising biosorbent for heavy metal removal in recent studies (Ningqin et al. 2020). Its large‐scale cultivability, low safety risks, and ease of handling make it an attractive option (Segal‐Kischinevzky et al. 2022; Stathatou et al. 2022). A wide range of yeast species have been explored for biosorption applications. These studies collectively highlight the potential of yeast as an effective and sustainable approach to heavy metal remediation.

This study investigates the biosorption of Cr(III) using *P. kudriavzevii* JD2 immobilized perlite. The main objective of the study was design material for simultaneously preconcentration and adsorption of Cr(III) ion. The selection of this novel adsorbent was based on its promising high adsorption capacity, eco‐friendly nature, low‐cost, and lack of prior use in heavy metal removal. Perlite, which was previously used only adsorbent alone, was used as support material for immobilization with *P. kudriavzevii* JD2. Acid‐treated and *P. kudriavzevii* JD2 immobilized perlite were characterized using SEM and TEM. The research encompasses a comprehensive examination of various parameters influencing the biosorption process, including pH of the analyte solution, eluent type and volume, flow rate, adsorbent mass, and the impact of interfering elements. Additionally, analytical parameters such as precision, kinetics, and thermodynamic studies were done. The results demonstrate the effective adsorption capacity of the prepared adsorbent, suggesting its potential application in water treatment processes for the removal of Cr(III).

## 2 Materials and Methods

### 2.1 Instrumentation

An Varian 249FS Model flame atomic adsorption spectroscopy (AAS) was used for the determination of Cr(III). A chrome hollow cathode lamp was used for analysis under the specified conditions: wavelength set at 357.9 nm, spectral bandwidth of 0.2 nm, lamp current of 7.0 mA, and acetylene flow rate of 2.90 L/min. The solution pH was adjusted with a digital Hannah pen pH meter (Model HI2211). For SPE experiments, a reservoir with a top that can hold 250 mL of solution and a glass column with a length of 10 cm and a width of 1 cm. The solutions flow rate was adjusted with peristaltic pump.

### 2.2 Preparation of Solutions and Materials

All solutions were prepared using distilled water. A standard Cr(III) solution was prepared through the dilution of a stock solution (1000 mg/L). Perlite samples were procured from Genper Mining Industry. To activate the perlite, it was treated with 1 and 2 mol/L H₂SO₄ and CH_3_COOH. The acid activated perlite was subsequently washed with distilled water and stored in polyethylene bottles.

### 2.3 Preparation of YPD Liquid Medium

A YPD (yeast extract‐peptone dextrose, Merck) liquid medium was employed for yeast cultivation. To prepare the medium, 20 g of D‐glucose, 10 g of yeast extract, and 20 g of peptone were accurately weighed and dissolved in distilled water using a magnetic stirrer. The final volume was adjusted to 1000 mL with distilled water, and the pH was maintained at 6.5 ± 0.2. The prepared medium was sterilized in an autoclave (Sanyo) at 121°C for 15 min.

### 2.4 Preparation of Dead Yeast Biomass

The *Pichia kudriavzevii* JD2 yeast strain used in this study was obtained from the stock culture collection of the Biotechnology Laboratory, Biology Department, Faculty of Science, Gazi University (Aakef and Yuksekdag 2023; Aakef 2018). A 2% inoculum of *P. kudriavzevii* JD2 yeast was added to 250 mL of YPD liquid medium and incubated at 37°C for 48 h. To enhance yeast growth, the inoculation and incubation process was repeated once more in fresh YPD medium at 30°C for 24 h with shaking at 200 rpm. The resulting yeast culture was centrifuged at 5000 rpm for 10 min. The supernatant was discarded, and pellet was washed repeatedly with sterile distilled water. Subsequently, the pellet was treated with 0.1 mol/L HCl for 10 min. Acid removal was achieved through multiple washes with sterile distilled water followed by centrifugation at 5000 rpm for 10 min. The obtained dead *P. kudriavzevii* JD2 biomass was divided into 500 μL aliquots and stored at −80°C overnight before lyophilization to obtain a powdered form (Aakef 2018).

### 2.5 Immobilization of Yeast on Perlite

Perlite was acid activated by keeping under reflux in a magnetic stirrer with 250 mL 2 mol/L H_2_SO_4_ (sulfuric acid) for 8 h at 97°C. And then, it was rinsed with distilled water. Presence of SO_4_^2−^ ion was tested by adding 5% BaCl_2_. When no white precipitate is observed, it was concluded that SO_4_^2−^ was completely removed. Activated samples were dried in a desiccator. Then, the immobilization was performed as follows: 1 g of activated perlite and 100, 200, 300, 400, and 500 mg of *Pichia kudriavzevii* were mixed. The mixtures were mixed with 2 mL distilled water. After mixing, it was dried in an oven for 30 min at 105°C. The wetting and drying procedure was repeated several times to maximaise the immobilization. The immobilized biomass was ground in a mortar with a pestle till fine powder was obtained.

### 2.6 Preparation of Column

Glass columns with a capacity of approximately 250 mL were used for this study. A layer of glass wool was placed at the bottom of each column, followed by 0.2 g of perlite immobilized with *Pichia kudriavzevii*. Another layer of glass wool was added on top of the adsorbent. Before use, the columns were thoroughly cleaned with distilled water to remove impurities. Subsequently, solutions of NaOH and HCl were passed through the columns to standardize the pH conditions.

### 2.7 Preconcentration Procedure

A SPE system was employed for the biosorption of Cr(III). Sample solutions containing 0.4 mg/L of Cr(III) were prepared and their pH was adjusted using HCl and buffer solutions of CH₃COOH/CH₃COONa and NaH₂PO₄/Na₂HPO₄. These sample solutions were passed through columns containing the adsorbent using a peristaltic pump. Subsequently, Cr(III) was eluted from the adsorbent with 2 mol/L HNO₃. The concentration of Cr(III) in the eluent was detected using atomic absorption spectroscopy (AAS). Perlite immobilized with *Pichia kudriavzevii* JD2 was packed into glass columns and reused after washing with 2 mol/L HNO₃ and distilled water.

## 3 Results and Discussion

### 3.1 Surface Characterization

**FIGURE 1:**
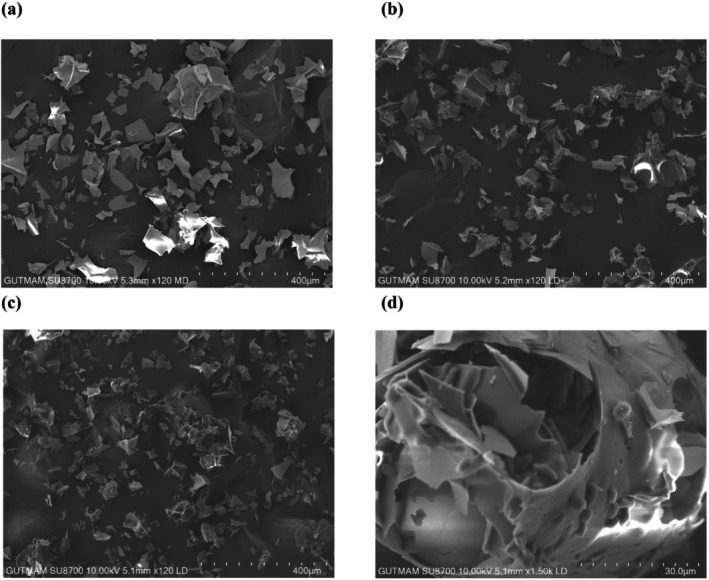
SEM images of (a) raw perlite, (b) acid activated with 2 mol/L H_2_SO_4_ perlite, and (c and d) acid activated with 2 mol/L H_2_SO_4_ immobilized on *Pichia kudriavzevii* JD2 yeast.

Raw perlite, acid activated with 2 mol/L H_2_SO_4_ perlite and acid activated with 2 mol/L H_2_SO_4_ immobilized on *Pichia kudriavzevii* JD2 yeast perlite were characterized by SEM. SEM images shown in Figure 1. According to SEM images, the surface porosity perlite samples activated with 2 mol/L H_2_SO_4_ (Figure 1b) and immobilized on *Pichia kudriavzevii* JD2 yeast (Figure 1c,d) are increased compared to raw perlite sample (Figure 1a).

### 3.2 Surface Area Analysis

The Brunauer, Emett, and Teller (BET) surface area results obtained by using nitrogen adsorption isotherms for raw and perlite treated with 1 and 2 mol/L H_2_SO_4_ and CH_3_COOH are given in Table 1.

**TABLE 1:** Surface area analysis of raw and acid activated with 1 and 2 mol/L H_2_SO_4_ and CH_3_COOH perlite.

Analysis	Raw perlite	1 mol/L H_2_SO_4_	2 mol/L H_2_SO_4_	1 mol/L CH_3_COOH	2 mol/L CH_3_COOH
Single point SA (m^2^/g)	7.99	8.84	11.99	7.49	8.89

The surface area of raw perlite at a single point was determined as 7.99 m^2^/g. As a result of the acid activation of perlite with 1 and 2 mol/L H_2_SO_4_ and CH_3_COOH acids, Al^3+^, Fe^3+^, Ca^2+^, Mg^2+^, Na^+^, K^+^ ions left behind and caused an increase of porosity in the specific surface area. It was observed that the surface area of acid activated perlite with 2 mol/L H_2_SO_4_ increased approximately two times (11.99 m^2^/g) compared to raw perlite (7.99 m^2^/g) and reached the highest value.

### 3.3 Optimization of Preconcentration Parameters

#### 3.3.1 Effect of Solution pH on the Recovery of Cr(III)

**FIGURE 2:**
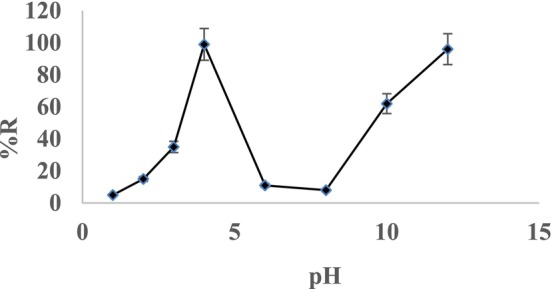
Effects of pH on the biosorption of Cr(III) 0.4 mg/L; eluent solution 2 mol/L HNO_3_ 10 mL.

The preconcentration of Cr(III) metal ions was investigated using the solid phase method by varying elution type, volume, pH, adsorbent dosage, sample solution flow rate, and the influence of interfering ions. To evaluate the effect of pH on Cr(III) recovery, experiments were conducted within a pH range of 1–12 while maintaining a constant sample volume of 25 mL. The recovery percentages of Cr(III) ions were determined for each pH value. The solutions with Cr(III) were passed through biosorbent placed on column with 1–3 mL/min flow rate. The results, depicted in Figure 2, indicate that the maximum recovery of Cr(III) ion was achieved at pH 4 and pH 12 with 100% of efficiency. In another study of adsorption Cr(III) by using activated carbon ion‐imprinted sorbent. The results are similar to our study, as optimum adsorption was performed in pH 4 (Rukiye, Canlidinc, and Orhan 2022).

#### 3.3.2 The Effect of the Immobilized Perlite and the Amount of *Pichia Kudriavzevii* Yeast on the Recovery Efficiency

**FIGURE 3:**
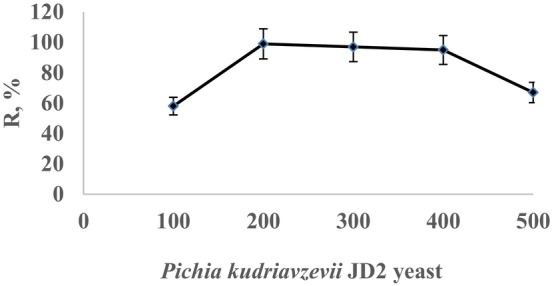
The effect of the amount of perlite and yeast immobilized on *Pichia kudriavzevii* JD2 yeast on the recovery efficiency.

To assess the impact of *Pichia kudriavzevii* JD2 yeast on recovery efficiency, varying amounts of yeast (100, 200, 300, 400, and 500 mg) were immobilized on 1 g of perlite. The effect of adsorbent dosage was investigated between 100 and 500 mg. Subsequently, 25 mL solutions containing 0.4 mg/L of Cr(III) were passed through the columns. The recovered Cr(III) ions were determined using AAS. The recovery results are presented in Figure 3. The optimal preconcentration conditions were determined to be 200 mg of *Pichia kudriavzevii* JD2 immobilized on 1 g of perlite, with a flow rate of 1–3 mL/min and a solution pH of 4. In similar research with recovery of Cu(II), Zn(II), and Cd(II) was used 200 mg of *Penicillium digitatum* immobilized on pumice stone. It was found optimum for preconcentration too as this study (Baytak et al. 2008).

#### 3.3.3 Effect of Recovery Solution Type and Volume

The influence of recovery solution type, volume, and concentration on Cr(III) ion recovery efficiency was investigated using model solutions. To elute the adsorbed Cr(III) from the column, 1, 2, and 3 mol/L solutions of HCl and HNO₃ were tested. Results indicated that 10 mL of 2 mol/L HNO₃ and 5 mL of 3 mol/L HNO₃ achieved 99% recovery of Cr(III) ions adsorbed on perlite‐immobilized *Pichia kudriavzevii* JD2 yeast. The flow rate through the columns during the recovery phase was maintained at 1–3 mL/min. Table 2 summarizes the recovery efficiencies obtained with different solution types and concentrations. All experiments were conducted at a constant flow rate of 1 mL/min and a solution pH of 4. In another literature on biosorption of Cr(III) ions on *Mucor pusillis* (Lindt, 1886) immobilized on sepiolite powder were used 5 mL 1% HNO_3_, 2 mL 5% HNO_3_ (Aakef 2018). In work mentioned before of adsorption of Cr(III) on carbon based ion‐imprinted sorbent an optimum eluent type and volume was 5 mL 4 mol/L HNO_3_ (Rukiye, Canlidinc, and Orhan 2022).

**TABLE 2:** Effect of volume and concentration of recovery solution.

Element	Eluent type	Volume (mL)	Concentratıon (mol/L)	Recovery (%)
Cr^3+^	HCl	10	1	74.2 ± 0.1
			2	73.0 ± 0.4
			3	80.0 ± 0.4
		5	1	26.0 ± 0.4
			2	30.4 ± 0.2
			3	52.0 ± 1.4
	HNO_3_	10	1	90.1 ± 0.1
			2	100.4 ± 0.3
			3	85.0 ± 0.6
		5	1	37.5 ± 0.1
			2	80.0 ± 0.5
			3	99.3 ± 0.5

#### 3.3.4 Effect of Flow Rate

**FIGURE 4:**
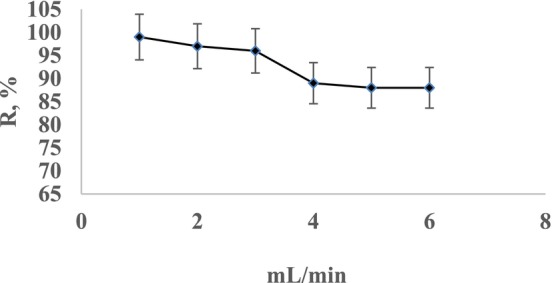
Effect of solution flow rate on recovery.

Study mentioned above in adsorption of Cr(III), the optimum used flow rate was 1–3 mL/min (Sıtkı and Abdul 2022). In another study of preconcentration of Pb(II), Ni(II), and Zn(II) on *Bacillus subtilis* loaded multiwalled carbon nanotube recoveries were 100% at 1 and 2 mL/min (Sadin et al. 2023). The biosorption efficiency of Cr(III) in the adsorbent is impacted by the flow rate. The rate of metal ion transfer to yeast binding sites is influenced by the flow rate of the solution. This is because the binding of metal from the solution to the perlite immobilized to the yeast *Pichia kudriavzevii* JD2 is affected by the flow rate of the solution. In the experiment, the flow rate was adjusted with a peristaltic pump in the range of 1–6 mL/min. The optimum recovery condition for Cr(III) solutions was found to be a flow rate of 1 mL/min. The effect of the flow rate on the recovery efficiency of Cr(III) ion is shown in Figure 4.

#### 3.3.5 Effect of the Volume of Sample Solution and Concentration on Recovery

**FIGURE 5:**
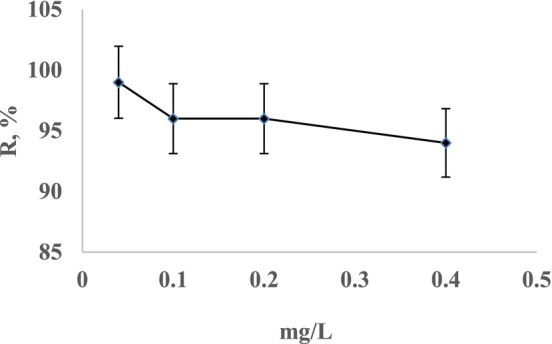
Effect of Cr(III) concentration on recovery.

To determine the biomass capacity, the recovery of Cr(III) at concentrations of 0.04 mg/L, 0.1, 0.2, and 0.4 mg/L was investigated. One milliliter of a 10 mg/L Cr(III) standard solution was diluted with pH‐adjusted solution to prepare 25, 50, 100, and 250 mL Cr(III) solutions. These solutions were passed through the columns under optimized conditions (pH, recovery solution type, flow rate). Despite the low concentrations of sample analytes, the recovery percentage remained consistent. The recovery results are shown in Figure 5. In different study of preconcentration of Cr(III) ions on activated carbon based ion‐imprinted sorbent solutions containing Cr(III) ions concentration range was 0.2–0.02 mg/L. As this study showed the preconcentration recovery dramatically decreased as the Cr(III) ion concentration decreased (Rukiye, Canlidinc, and Orhan 2022).

#### 3.3.6 Effect of Column Reuse

**FIGURE 6:**
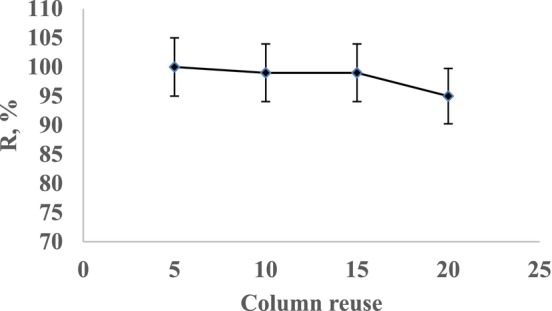
Effect of column reuse on Cr(III) ion recovery.

To assess the reusability of the biomass, the sample solution was passed through and recovered from the column 20 times. The changes in recovery capacity were monitored by analyzing the analytes. The analyte passing and recovery efficiencies of the columns were evaluated. When not in use, the columns were filled with distilled water. The impact of repeated column use on the recovery efficiency of perlite immobilized with *Pichia kudriavzevii* JD2 yeast is illustrated in Figure 6. In a study, preconcentration of Cr(III) ions on activated carbon based ion‐imprinted sorbent results showed significant decrease at least after 50 cycles or more (Rukiye, Canlidinc, and Orhan 2022).

#### 3.3.7 Effect of Interfering Ions on Recovery of Cr(III)

The effect of the interfering elements was investigated by adding alkaline and alkaline earth elements to a sample solution containing 0.4 mg/L Cr(III) ion. Interfering metal ions concentration was 1–25 mg/L range. The solutions passed through columns under the most optimum conditions (pH 4, flow rate 1 mL/min, and recovery solution 2 mol/L 10 mL HNO_3_). The preconcentrated Cr(III) ions are determined by AAS. As a result of the experiments, it was shown that the concentrations of Mg^2+^, Ca^2+^, Fe^2+^, Cd^2+^, Pb^2+^ up to 10 mg/L, K^+^, Cu^2+^, Ni^2+^, Zn^2+^ up to 25 mg/L, Mn^2+^, Co^2+^ up to 1 mg/L did not interfere with the recovery efficiency of Cr(III) ions. Two milliliter of 0.01 M EDTA solution was used to eliminate the alkali and alkaline soil interference affecting Cr(III) ions. The results are given in Table 3. Effect of interfering ions on adsorption of Cr(III) ions on multiwalled carbon nanotubes conducted by testing various ions and pose no interference in preconcentration of Cr(III) ions (Yu et al. 2012).

**TABLE 3:** Effect of interfering ions on recovery of Cr(III).

Interfering ion	Concentration (mg/L)	Recovery (%) Cr(III)
K^+^	1	99.0 ± 1.0
	5	99.0 ± 1.0
	10	97.0 ± 1.0
	25	99.0 ± 0.3
Mg^2+^	1	99.8 ± 0.1
	5	99.2 ± 0.3
	10	99.7 ± 0.4
	25	32.7 ± 0.1
Ca^2+^	1	99.3 ± 0.1
	5	99.3 ± 0.1
	10	99.5 ± 0.1
	25	19.7 ± 0.1
Cu^2+^	1	99.5 ± 0.1
	5	99.0 ± 0.2
	10	99.8 ± 0.2
	25	99.4 ± 0.1
Cd^2+^	1	99.0 ± 0.1
	5	99.5 ± 0.2
	10	99.1 ± 0.4
Mn^2+^	1	95.5 ± 0.1
	5	42.5 ± 0.1
	10	48.6 ± 0.3
Pb^2+^	1	99.2 ± 0.1
	5	99.2 ± 0.1
	10	99.4 ± 0.1
	25	34.6 ± 0.1
Ni^2+^	1	99.4 ± 0.1
	5	99.2 ± 0.1
	10	99.1 ± 0.1
	25	99.4 ± 0.1
Zn^2+^	1	99.9 ± 0.1
	5	99.4 ± 0.2
	10	99.1 ± 0.5
	25	99.9 ± 0.1
Fe^3+^	1	99.8 ± 0.2
	5	99.6 ± 0.1
	10	99.6 ± 0.2
	25	72.8 ± 0.2
Co^2+^	1	99.8 ± 0.1
	5	78.6 ± 0.2

### 3.4 Analytical Features

Analytical parameters such as limits of detection (LOD) and quantification (LOQ) were detected for Cr(III) under optimum conditions. To designate the detection limit, 50 mL solutions containing 0.1 mg/L Cr(III) or not containing Cr(III) ion were prepared. The solutions were passed through columns over perlite that was acid activated with 2 mol/L H_2_SO_4_ immobilized on *Pichia kudriavzevii* JD2 yeast under the most suitable conditions, pH 4, and the flow rate was adjusted to 3 mL/min. The most suitable recovery solution determined for adsorbent Cr(III) ion was recovered using 50 mL of 2 mol/L HNO_3_. The recovery solution was read 30 times, and the standard deviation of the obtained absorbance values was calculated. While calculating the detection limit for Cr(III), it was divided by the obtained enrichment coefficient. The results of LOD and LOQ are shown in Table 4.

**TABLE 4:** The results of limits of LOD and LOQ.

Element	Limits of detection (LOD), μg/L	Limits of quantification (LOQ), μg/L
Cr(III)	4.8	15.8

#### 3.4.1 Determination of Cr(III) in Real Samples and Accuracy of the Method

Cr(III) ions determination was made in Ankara laboratory tap water, mineral water 1 and mineral water 2. Ankara tap water sample was taken from the laboratory fountain, mineral water 1—Ceysu mineral water and mineral water 2—Pursu mineral water. Enrichment was carried out in the most suitable conditions specified for Cr(III) determination and Cr(III) ion was determined (Table 5).

**TABLE 5:** Determination of Cr(III) ions in various water samples immobilized on *Pichia kudriavzevii* JD2 yeast with 2 mol/L H_2_SO_4_ and acid activated perlite.

Water samples	Element	Added (mg/L)	Found	Relative error (%)
Tap water	Cr(III)	—	ND	—
		0.4	0.38 ± 0.01	−5.0
		0.8	0.76 ± 0.06	−5.0
Mineral water 1		—	ND	—
		0.4	0.43 ± 0.01	7.5
		0.8	0.83 ± 0.02	3.8
Mineral water 2		—	ND	—
		0.4	0.42 ± 0.04	5.0
		0.8	0.86 ± 0.02	7.5

To investigate the accuracy of the method, Cr(III) determination was performed in the standard reference material (wastewater sample). For this, standard reference material (wastewater) (SPS‐WW1 Batch 109) was used. The standard reference material was passed through the column under the most suitable conditions with pH 4 and recovery solution 2 mol/L 10 mL HNO_3_. The obtained result is shown in Table 6.

**TABLE 6:** Determination of Cr(III) in the standard reference material (SPS‐WW1 Batch 109).

Element	Concentration (mg/L)		Relative error, %
	Certified	Found	
Cr(III)	0.500	0.457 ± 0.006	−8.6

*Note*: The concentration of elements in wastewater sample mg/L: Al: 2, As: 0.1, Cd: 0.02, Co: 0.06, Cr: 0.2, Cu: 0.4, Fe: 1, Mn: 0.4, Ni: 1, P: 1, Pb: 0.1, V: 0.1, Zn: 0.6.

Additionally, the important parameters are comparatively presented in Table 7. These studies collectively highlight the potential of yeast as an effective and sustainable approach to heavy metal remediation.

**TABLE 7:** Literature comparison in view of analytical parameters.

Adsorbent	LOD (μg/L)	Detection technique	Conditions	Reference
Raw and acid treated white colored calcium‐bentonite adsorption of Cr(III)		ICP‐OES	pH 1.0–3.5, temperature 40°C–60°C	Cinar Acar and Yuksekdag (2023a)
Solid phase extraction method using activated carbon based ion‐imprinted sorbent	Cr(III): 34	FAAS	pH 4, eluent 5 mL 4 mol/L HNO_3_	Rukiye, Canlidinc, and Orhan (2022)
Biosorption onto *Mucor pusillis* (Lindt, 1886) immobilized on sepiolite powder	Co(II): 1.2, Cr(III): 1.3, Mn(II): 1.4, Ni(II) 0.6, Zn(II): 0.2	ICP‐AES	pH 6, eluent: 5 mL 1% HNO_3_, 2 mL 5% HNO_3_, flow rate 1 mL/min	Sıtkı and Abdul (2022)
Preconcentration with magnetic nanoparticles	Cr(III): 0.335	AAS	pH 8, eluent 2 mol/L HCl, extraction time 5 min, adsorbent dosage 25 mg	Caylak (2024)
Solid phase extraction method using coffee siverskin	Cr(III): 6	FAAS	pH 2, eluent 0.2 mL 0.1 mol/L HCl, adsorbent amount 25 mg	Silva et al. (2022)
Solid phase microextraction by activated charcoal molybdenum (IV) selenide—magnetite composite	Cr(VI): 0.36	FAAS	pH 2.5, eluent 2 mL 3 mol/L HNO_3_, adsorbent mass 20 mg	Zeliha et al. (2024)
Solid phase extraction using modified magnetite nanoparticles	Cr(VI): 0.083	FAAS	pH 7, sample volume 350 mL, adsorbent amount 100 mg, eluent 2 mL ethanol	Karimi et al. (2013)
Green alga *Chlorella sorokiniana* immobilized in loofa sponge	—	AAS	pH 4, 0.2 mol/L HNO_3_	Nasreen et al. (2008)
*Pichia kudriavzevii* JD2 yeast immobilized on 2 mol/L H_2_SO_4_ acid activated perlite	Cr(III): 4.8	AAS	pH 4, 10 mL 2 mol/L HNO₃ and 5 mL of 3 mol/L HNO_3_, flow rate 1–3 mL/min	In this study

### 3.5 Adsorption Kinetics and Thermodynamic Parameters

**FIGURE 7:**
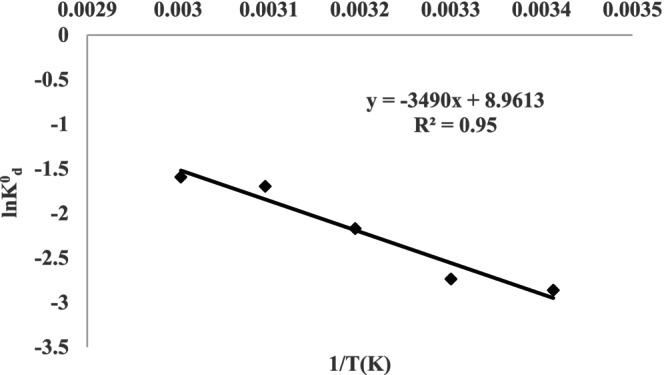
1/T (*K*) and ln *K* ^0^_*d*_ change graph in the adsorption of Cr(III) on acid activated with 2 mol/L H_2_SO_4_ perlite immobilized on *Pichia kudriavzevii* JD2 yeast.

Perlite, acid activated with 2 mol/L H_2_SO_4_ immobilized on *Pichia kudriavzevii* JD2 yeast, was shaken in a water bath at different temperatures for Cr(III) ion adsorption. The calibration curve drawn between 1/*T* (*K*) and ln *K* ^0^_*d*_ regarding adsorption is given in Figure 7 and the calculated thermodynamic data are given in Tables 7 and 8 (Azam et al. 2021).

**TABLE 8:** Thermodynamic data of adsorption of Cr(III) ions on acid activated with 2 mol/L H_2_SO_4_ perlite immobilized on *Pichia kudriavzevii* JD2 yeast.

Temperature (*K*)	*K* ^0^_*d*_	∆*G* ^0^ (kJ/mol)	∆*H* ^0^ (kJ/mol)	∆*S* ^0^ (J/mol K)	*E*_a_ (kJ/mol)
293	0.05	+7.2	+29.01	+74.5	−29
303	0.06	+6.4			
313	0.12	+5.6			
323	0.18	+4.9			

### 3.6 Adsorption Kinetics

**FIGURE 8:**
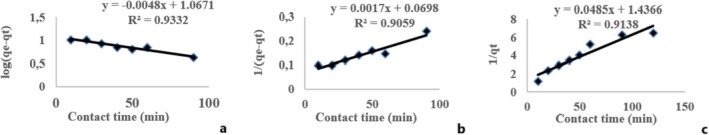
(a) Lagergren pseudo‐first‐order kinetic graph in the adsorption of Cr(III) onto acid activated with 2 mol/L H_2_SO_4_ perlite immobilized on *Pichia kudriavzevii* JD2 yeast, (b) pseudo‐second‐order kinetic graph, and (c) second‐order kinetic graph.

Adsorption kinetics of Cr(III) was determined on acid activated perlite with 2 mol/L H_2_SO_4_ immobilized on *Pichia kudriavzevii* JD2 yeast. Fifty microliter of 200 mol/L Cr(III) ion solution with 0.05 g of adsorbent were stirred at various time and temperature in water bath. Lagergren's pseudo‐first‐order, pseudo‐second‐order, and second‐order kinetic models were applied. The linearized form of the Lagergren's pseudo‐first‐order, pseudo‐second‐order, and second‐order kinetic models was given in Figure 8 (Azam et al. 2021).

In another study on adsorption of Cr(III), the correlation coefficient of pseudo‐second order kinetics model is higher than pseudo‐first‐order kinetics model. It is suggesting that pseudo‐second‐order can be best description for Cr(III) adsorption as this study too (Peng et al. 2020) (Table 9).

**TABLE 9:** Lagergren parameters of the adsorption of Cr(III) on acid activated perlite with 2 mol/L H_2_SO_4_ immobilized on *Pichia kudriavzevii* JD2 yeast.

Temperature	Pseudo‐first‐order			Pseudo‐second‐order			Second‐order		
	*q*_e_ (mg/g)	*k*_1_ (min^−1^)	*R* ^2^	*q*_e_ (mg/g)	*k*_2_ (g/min mg)	*R* ^2^	*q*_e_ (mg/g)	*k* (g/min mg)	*R* ^2^
293	3.6	−3.6 × 10^−4^	0.91	16.9	1.1 × 10^−2^	0.97	58.1	1.4 × 10^−2^	0.81
303	3.8	−1.7 × 10^−5^	0.87	15.7	2.8 × 10^−2^	0.98	17.9	−1.0 × 10^−4^	0.89
313	2.9	−4.0 × 10^−5^	0.93	20.9	1.8 × 10^−3^	0.91	14.3	1.7 × 10^−3^	0.91
323	3.6	−3.5 × 10^−5^	0.83	27.7	1.0 × 10^−3^	0.99	19.8	7.0 × 10^−4^	0.82
333	4.1	−6.5 × 10^−5^	0.91	24.8	1.6 × 10^−3^	0.91	33.2	1.4 × 10^−3^	0.92

### 3.7 Cr(III) Adsorption Studies on 2 mol/L H_2_SO_4_ Activated Perlite

**FIGURE 9:**
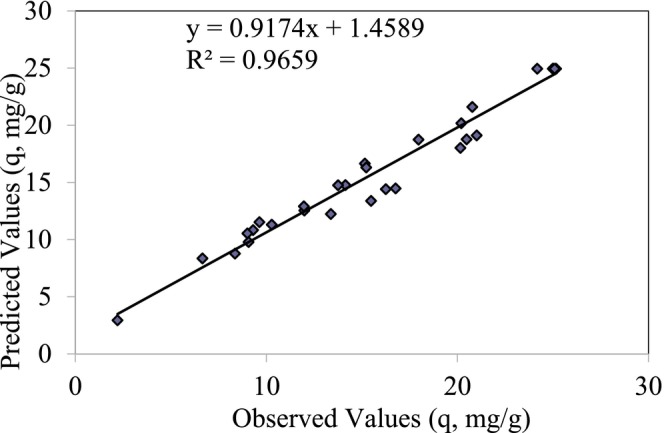
Relationship between experimental results and predicted results.

A central composite design (CCD) was employed to optimize the batch adsorption of Cr(III) onto perlite activated with 2 mol/L H₂SO₄ from synthetic aqueous solutions. Four independent variables were investigated: pH (*X*₁), temperature (*X*₂), Cr(III) concentration (*X*₃), and contact time (*X*₄). The experimental data obtained from the CCD with variable ranges and levels as outlined in Table 10 were subjected to regression analysis using the least squares method to determine the optimal conditions for Cr(III) adsorption. Figure 9 illustrates the correlation between the experimental and predicted results. The experiment of adsorption behavior of Cr(III) were performed by adding 0.05 g of 2 mol/L H_2_SO_4_ activated perlite in 10 mL of a different concentrating of synthetic Cr(III) solution (50, 100, 150, 200, and 250 mg/L). The experiment was performed in a bath shaker (Cinar Acar and Yuksekdag 2023a).

**TABLE 10:** An experimental design was created according to the CCD model, with four independent variables for Cr(III) adsorption using 2 mol/L H_2_SO_4_ activated perlite.

No	Codded values				Response values *q* (mg/g)		
	*X*_1_	*X*_2_	*X*_3_	*X*_4_	Experimental values	Predicted values	Residual values
1	1	1	1	1	17.97	18.74	−0.77
2	1	1	1	−1	14.14	14.76	−0.62
3	1	1	−1	1	9.31	10.83	−1.52
4	1	1	−1	−1	9.01	10.53	−1.52
5	1	−1	1	1	15.16	16.65	−1.49
6	1	−1	1	−1	11.99	12.54	−0.55
7	1	−1	−1	1	8.36	8.77	−0.41
8	1	−1	−1	−1	6.66	8.35	−1.69
9	−1	1	1	1	20.78	21.60	−0.82
10	−1	1	1	−1	15.23	16.31	−1.08
11	−1	1	−1	1	11.97	12.91	−0.94
12	−1	1	−1	−1	10.29	11.31	−1.01
13	−1	−1	1	1	20.21	20.18	0.03
14	−1	−1	1	−1	13.76	14.75	−0.99
15	−1	−1	−1	1	9.64	11.53	−1.89
16	−1	−1	−1	−1	9.07	9.79	−0.72
17	2	0	0	0	16.77	14.48	2.29
18	−2	0	0	0	20.48	18.77	1.71
19	0	2	0	0	20.16	18.02	2.14
20	0	−2	0	0	16.26	14.41	1.85
21	0	0	2	0	13.38	12.24	1.14
22	0	0	−2	0	2.21	2.94	−0.73
23	0	0	0	2	21.01	19.11	1.90
24	0	0	0	−2	15.48	13.39	2.09
25	0	0	0	0	24.18	24.94	−0.76
26	0	0	0	0	25.14	24.94	0.20
27	0	0	0	0	25.17	24.94	0.23
28	0	0	0	0	25.01	24.94	0.07
29	0	0	0	0	25.03	24.94	0.09
30	0	0	0	0	24.99	24.94	0.05
31	0	0	0	0	25.09	24.94	0.15

The ANOVA result table of the regression model of Cr(III) adsorption on 2 mol/L H_2_SO_4_ activated perlite adsorbent is given in Table 11.

**TABLE 11:** ANOVA results in table of the regression model of Cr(III) adsorption onto perlite adsorbent activated with 2 mol/L H_2_SO_4_.

	df	SS	MS	Model *F*	Significance *F*
Regression	14	1211.70	86.55	25.51	2.72 × 10^−8^
Difference	16	54.27	3.39		
Total	30	1265.98			

*Note*: Multiple *R* = 0.978, *R* ^2^ = 0.97, adjustable *R* ^2^ = 0.920.

ANOVA (analysis of variance) analysis of the applied model within the 95% confidence interval, the compatibility of the model and experimental findings was examined (Oskui, Aghdasinia, and Sorkhabi 2019). A significant *F* value (*p* < 0.05) indicates that the model is statistically significant at the 95% confidence level. The regression was found to be statistically significant as the significance *F* value of the model was 2.72 × 10^−8^ (*p* < 0.05), and the model *F* value was 25.51. The correlation coefficient (*R* ^2^) value being determined as 95.7% exhibits that there is a high compatibility between the observed values and the predicted values.

The full polynomial equation obtained by the Student's *t*‐test shown on the coded values achieved from Cr(III) adsorption with the experimental design method is given in Equation (1).
(1)

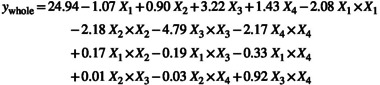




The polynomial equation showing the statistical significance levels of the coefficients determined as a result of the regression analysis using the *p* values obtained with the *t*‐test is given in Equation (2).

(2) $$\begin{array}{l} y_{\text{significant}}=24.94–1.07\;X_{1}+0.90\;X_{2}+3.22\;X_{3}+1.43\;X_{4}–2.08\;X_{1}\times X_{1}\\ –2.18\;X_{2}\times X_{2}–4.79\;X_{3}\times X_{3}–2.17\;X_{4}\times X_{4}–0.33\;X_{1}\times X_{4}+0.92\;X_{3}\times X_{4} \end{array}$$

Regression analysis was performed using the *p* values determined by the significance levels of the independent variables on the system in the polynomial equation. High “*t*” value (*t* > 2.12) and low “*p*” value (*p* < 0.05) are interpreted as increasing the significance value of the coefficient of the variables.

In line with the results obtained from experimental data (Table 12), it is seen that temperature, initial Cr(III) concentration, and contact time are important factors that have an increasing effect on adsorption, while pH has a decreasing effect. While it was determined that only the initial Cr(III) concentration and contact time were significant among the binary interactions, the other binary interactions did not show a statistically significant effect depending on the *t* and high *p* value.

**TABLE 12:** Coefficients, *t*, and *p* values of regression analysis of Cr(III) adsorption.

	Coefficients	Standard error	*t* stat	*p*
Intersection	24.94	0.70	35.83	7.34 × 10^−17^ *
*X*_1_	−1.07	0.38	−2.86	9.00 × 10^−3^ *
*X*_2_	0.90	0.38	2.40	1.82 × 10^−2^ *
*X*_3_	3.22	0.38	8.56	1.72 × 10^−7^ *
*X*_4_	1.43	0.38	3.80	1.49 × 10^−3^ *
*X*_1_ *X*_1_	−2.08	0.34	−6.04	1.63 × 10^−5^ *
*X*_2_ *X*_2_	−2.18	0.34	−6.34	1.10 × 10^−5^ *
*X*_3_ *X*_3_	−4.79	0.34	−13.90	1.76 × 10^−10^ *
*X*_4_ *X*_4_	−2.17	0.34	−6.31	7.50 × 10^−6^ *
*X*_1_ *X*_2_	0.17	0.46	0.36	8.57 × 10^−1^
*X*_1_ *X*_3_	−0.19	0.46	−0.42	5.22 × 10^−1^
*X*_1_ *X*_4_	−0.33	0.46	−0.71	4.01 × 10^−1^
*X*_2_ *X*_3_	0.01	0.46	0.02	8.57 × 10^−1^
*X*_2_ *X*_4_	−0.03	0.46	−0.07	8.97 × 10^−1^
*X*_3_ *X*_4_	0.92	0.46	2.00	4.52 × 10^−2^ *

*Note: X*_1_: pH, *X*_2_: temperature, *X*_3_: initial Cr(II) concentration, *X*_4_: contact time.

* Statistically significant.

In the study where Cr(III) adsorption on acid activated perlite was examined by experimental design method, it was shown that the model was statistically significant by regression analysis using the significance *F* value and model *F* value.

#### 3.7.1 Single Effects of Significant Parameters Affecting Cr(III) Adsorption

**FIGURE 10:**
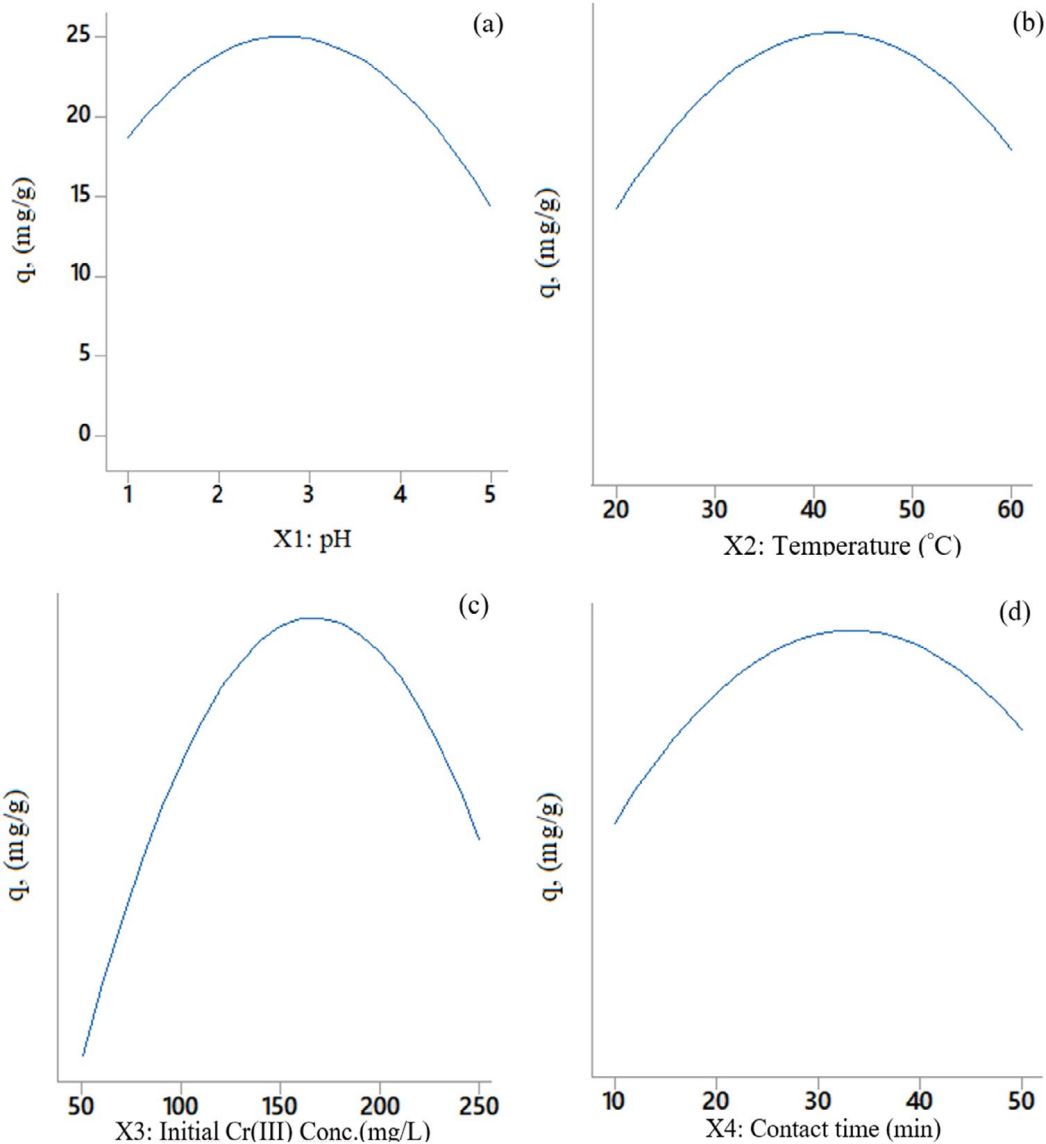
Effect of main parameters at Cr(III) adsorption onto 2 mol/L H_2_SO_4_ acid activated perlite.

The effect of solution pH on Cr(III) adsorption on acid activated perlite with 2 mol/L H_*2*_SO_4_ was increased in the pH range of 1.00–2.75 and decreased in the pH range of 2.75–5.0 (Figure 10a). As another study suggests, the adsorption of Cr(III) performed at pH around 5, stating that Cr(III) will precipitate after pH 5 (Guimarães et al. 2020). The maximum Cr(III) adsorption was achieved as a *Q* value of 25.8 mg/g under pH 2.7 condition. In temperature effect, there is an increase in Cr(III) adsorption at 20°C–40°C, a decrease is observed at 40°C–60°C. An increase in adsorption efficiency was also determined due to the formation of new active sites on the adsorbent surface with the increase in temperature. The maximum Cr(III) adsorption was achieved as a *Q* value of 25.0 mg/g at 42°C temperature (Figure 10b). In initial Cr(III) concentration effect, there is an increase in Cr(III) adsorption at 50–170 mg/L, a decrease is observed at 170–250 mg/L. The increase in the amount of Cr(III) adsorbed in milligrams per gram of adsorbent, depending on the enhancing Cr(III) concentration. The maximum Cr(III) adsorption was achieved at 167 mg/L Cr(III) concentration conditions with a *Q* value of 25.5 mg/g (Figure 10c). It was determined that contact time effect of the adsorption of Cr(III) on perlite activated with 2 mol/L H_2_SO_4_ increased in the contact time range of 10–34 min and decreased in the range of 35–50 min (Figure 10d). The maximum Cr(III) adsorption *Q* value was obtained as 25.2 mg/g under the conditions of contact time of 33 min.

#### 3.7.2 The Combined Effects of Significant Parameters Affecting Cr(III) Adsorption

**FIGURE 11:**
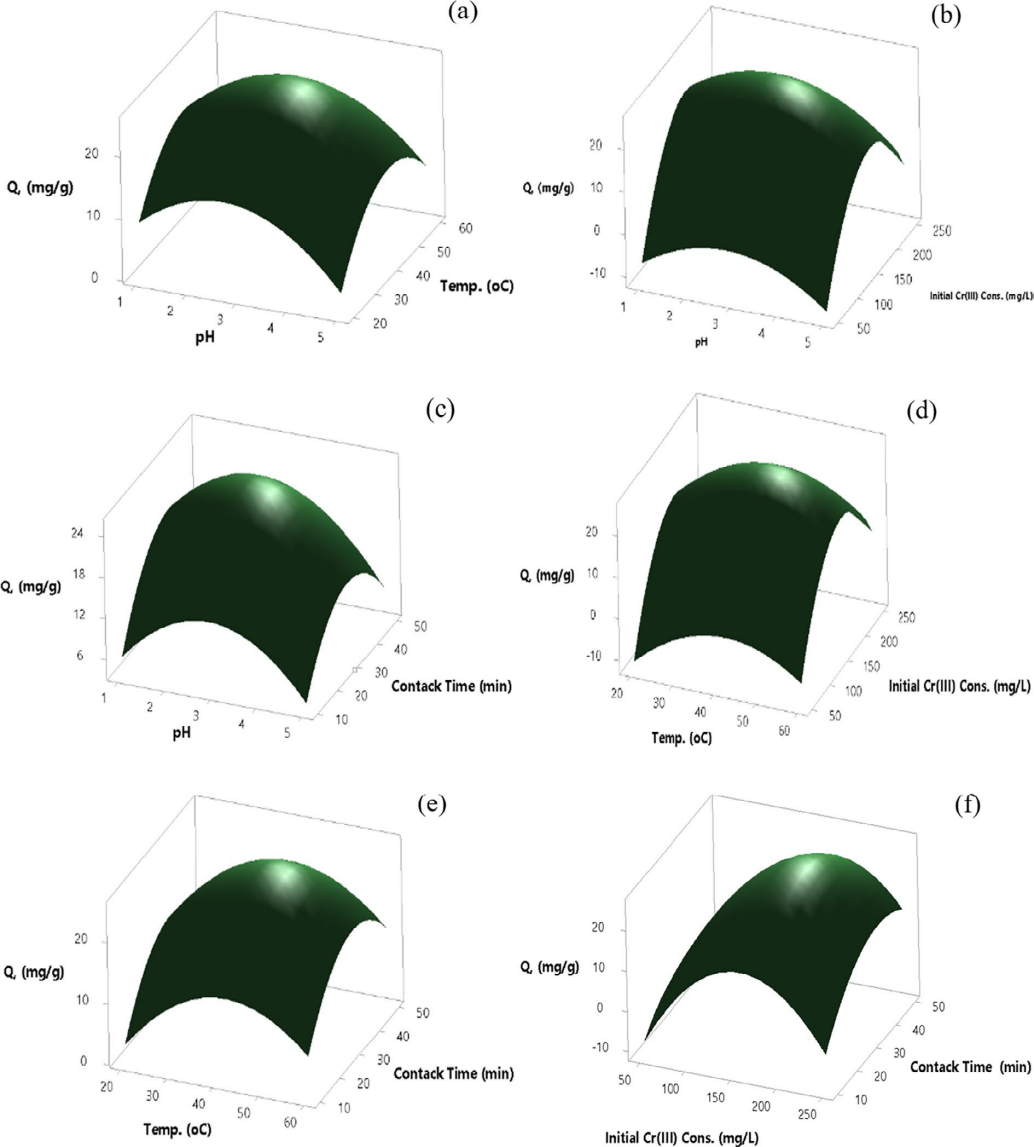
Response surface graphs: (a) pH and temperature, (b) pH and initial Cr(III) concentration, (c) pH and contact time, (d) temperature and initial Cr(III) concentration, (e) temperature and contact time, (f) initial Cr(III) concentration and contact time.

The combined effects of significant parameters affecting Cr(III) adsorption were exhibited in 3D surface plots with factors at fixed levels (Figure 11a–f). The maximum value was achieved as 25.8 mg/g at an initial Cr(III) concentration of 169 mg/L and 34 min conditions (Figure 13f). Changes in binary interactions pH and temperature, initial Cr(III) concentration and contact time, and temperature and initial Cr(III) concentration, contact time did not have statistically significant effects on adsorption (Figure 11a–e).

**FIGURE 12:**
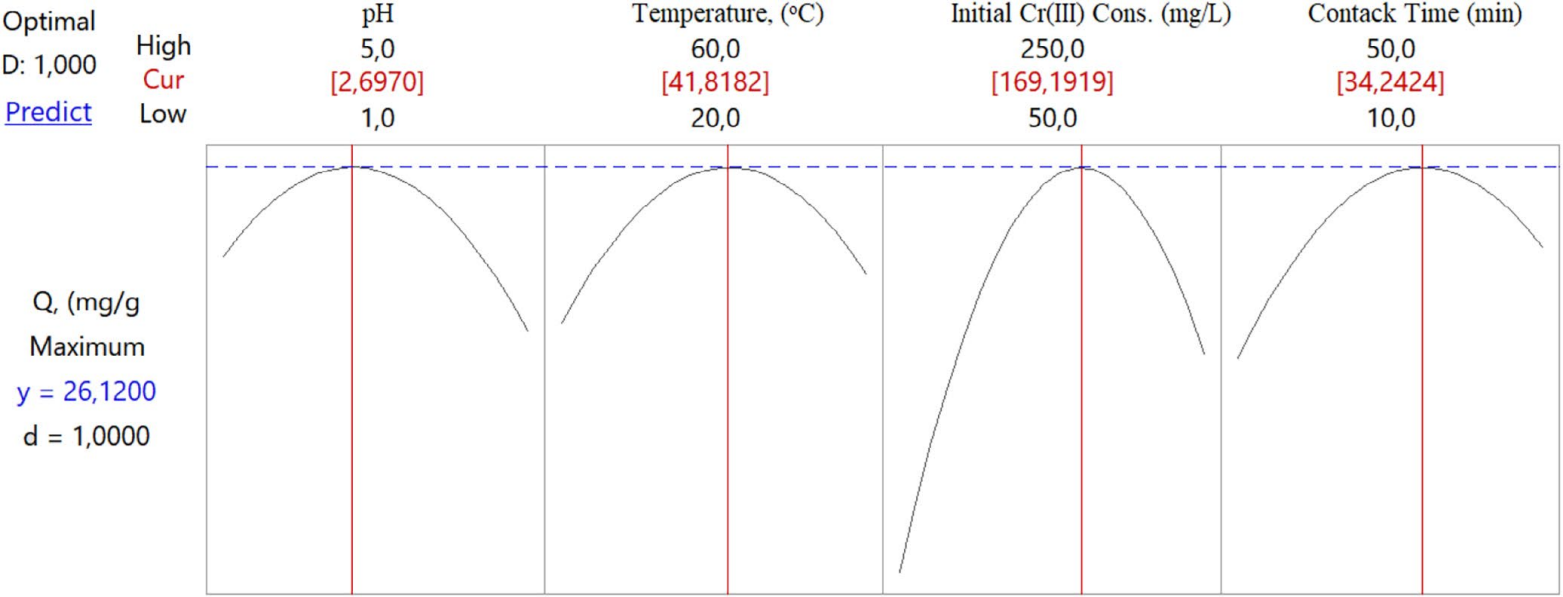
Optimum values of parameters according to CCD model with four independent variables for Cr(III) adsorption onto perlite activated with 2 mol/L H_2_SO_4_.

*Q* values in mg of Cr(III) adsorbed per gram of adsorbent were determined under optimum conditions (pH: 2.70, temperature: 41.8°C, initial Cr(III) concentration: 169.2 mg/L, and contact time: 34.2 min) were determined to be relatively low (26.12 mg/g) (Figure 12).

#### 3.7.3 Cr(III) Biosorption Studies of Pichia Kudriavzevii JD2 Adsorbent Immobilized With Perlite

**FIGURE 13:**
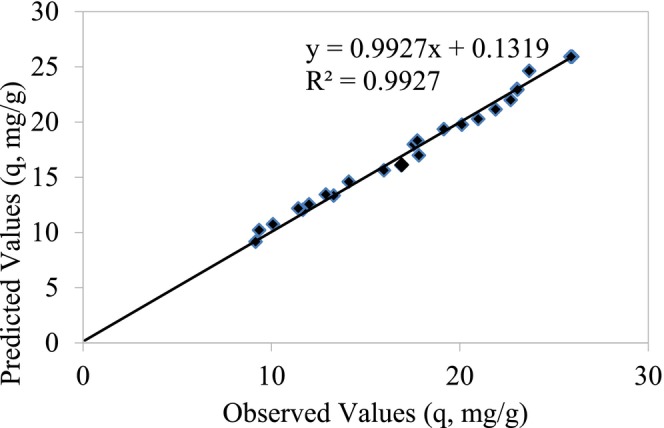
Relationship between experimental values and predicted values.

Using *Pichia kudriavzevii* JD2 (biosorbent) immobilized on perlite from synthetic aqueous solutions containing Cr(III), the CCD model of Cr(III) removal by batch method adsorption method was used and four different independent variables [pH of the solution] were determined with seven repetitions at the central points (*X*_1_), temperature (*X*_2_), Cr(III) concentration (*X*_3_), and contact time (*X*_4_). For Cr(III) adsorption experiments, regression was performed using the least squares method with the data obtained from the experimental design with the variable ranges and levels given in Table 13 and the independent variables of Cr(III) adsorption. Figure 13 shows the graph of the calibration between the experimental results and the predicted results.

**TABLE 13:** An experimental design was created according to the CCD model with four independent variables for adsorption of Cr(III) onto acid activated with 2 mol/L H_2_SO_4_ perlite immobilized on *Pichia kudriavzevii* JD2 yeast.

No	Codded values				Response values *q* (mg/g)		
	*X*_1_	*X*_2_	*X*_3_	*X*_4_	Experimental values	Predicted values	Residual values
1	1	1	1	1	23.04	23.02	0.02
2	1	1	1	−1	17.56	17.98	−0.42
3	1	1	−1	1	11.67	12.07	−0.40
4	1	1	−1	−1	10.09	10.75	−0.66
5	1	−1	1	1	19.15	19.36	−0.21
6	1	−1	1	−1	14.11	14.60	−0.49
7	1	−1	−1	1	9.35	10.22	−0.88
8	1	−1	−1	−1	9.16	9.17	−0.02
9	−1	1	1	1	23.68	24.64	−0.96
10	−1	1	1	−1	20.11	19.77	0.34
11	−1	1	−1	1	13.30	13.35	−0.05
12	−1	1	−1	−1	11.42	12.19	−0.77
13	−1	−1	1	1	23.06	22.94	0.12
14	−1	−1	1	−1	17.75	18.33	−0.58
15	−1	−1	−1	1	12.89	13.45	−0.56
16	−1	−1	−1	−1	11.99	12.56	−0.56
17	2	0	0	0	16.91	16.14	0.77
18	−2	0	0	0	21.90	21.15	0.75
19	0	2	0	0	20.98	20.28	0.69
20	0	−2	0	0	17.83	17.00	0.83
21	0	0	2	0	15.97	15.63	0.33
22	0	0	−2	0	0.10	−1.09	1.18
23	0	0	0	2	22.70	22.00	0.70
24	0	0	0	−2	16.90	16.08	0.82
25	0	0	0	0	25.88	25.92	−0.04
26	0	0	0	0	25.95	25.92	0.03
27	0	0	0	0	25.87	25.92	−0.05
28	0	0	0	0	25.96	25.92	0.03
29	0	0	0	0	25.92	25.92	0.00
30	0	0	0	0	25.97	25.92	0.05
31	0	0	0	0	25.91	25.92	−0.02

The ANOVA result table of the regression model of Cr(III) adsorption on *Pichia kudriavzevii* JD2 biosorbent immobilized on perlite is given in Table 14.

**TABLE 14:** ANOVA result in table of the regression model of Cr(III) adsorption on the biosorbent.

	df	SS	MS	Model *F*	Significance *F*
Regression	14	1289.99	92.14	156.21	2.24 × 10^−14^
Difference	16	9.44	0.59		
Total	30	1299.43			

*Note*: Multiple *R* = 0.978, *R* ^2^ = 0.957, adjustable *R* ^2^ = 0.920.

ANOVA analysis of the applied model within the 95% confidence interval was investigated and the concordance of the model and experimental results was examined (Oskui, Aghdasinia, and Sorkhabi 2019). The model was found to be statistically significant at the 95% confidence level, as evidenced by the highly significant *F* value (*p* < 0.05, *F* = 156.21). The high correlation coefficient (*R* ^2^ = 99.3%) indicates excellent agreement between the observed and predicted values.

The full polynomial equation obtained by the Student's *t*‐test shown on the coded values derived from Cr(III) biosorption experiments with the experimental design method is given in Equation (3).
(3)

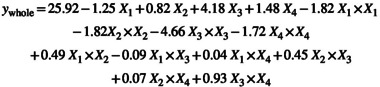




The polynomial equation showing the statistical significance levels of the coefficients determined as a result of the regression analysis using the *p* values obtained with the *t*‐test is given in Equation (4).

(4) $$\begin{array}{l} Y_{\text{significant}}=25.92–1.25\;X_{1}+0.82\;X_{2}+4.18\;X_{3}+1.48\;X_{4}–1.82\;X_{1}\times X_{1}–1.82\\ X_{2}\times X_{2}–4.66\;X_{3}\times X_{3}–1.72\;X_{4}\times X_{4}+0.49\;X_{1}\times X_{2}+0.45\;X_{2}\times X_{3}+0.93\;X_{3}\times X_{4} \end{array}$$

Regression analysis was performed using the *p* values determined by the significance levels of the independent variables on the system in the polynomial equation. The high “*t*” value (*t* > 2.12) and low “*p*” value (*p* < 0.05) are interpreted as increasing the significance value of the coefficient of the variables.

In line with the results obtained from experimental data (Table 15), it is seen that temperature, initial Cr(III) concentration, and contact time are the main factors that have an increasing effect on biosorption, while pH has a decreasing effect. Among the binary interactions, it was determined that pH and temperature, temperature and initial Cr(III) concentration, and initial Cr(III) concentration and contact time had significant and increasing effects. It was designated that other binary interactions did not show a statistically significant effect depending on the *t* and high *p* value.

**TABLE 15:** Coefficients, *t*, and *p* values of regression analysis of Cr(III) adsorption.

	Coefficients	Standard error	*t* stat	*p*
Intersection	25.92	0.29	89.30	5.28 × 10^−23^ *
*X*_1_	−1.25	0.16	−7.99	5.63 × 10^−7^ *
*X*_2_	0.82	0.16	5.24	8.09 × 10^−5^ *
*X*_3_	4.18	0.16	26.66	1.09 × 10^−14^ *
*X*_4_	1.48	0.16	9.45	5.99 × 10^−8^ *
*X*_1_ *X*_1_	−1.82	0.14	−12.67	9.33 × 10^−10^ *
*X*_2_ *X*_2_	−1.82	0.14	−12.67	9.28 × 10^−10^ *
*X*_3_ *X*_3_	−4.66	0.14	−32.46	4.95 × 10^−16^ *
*X*_4_ *X*_4_	−1.72	0.14	−11.98	2.11 × 10^−9^ *
*X*_1_ *X*_2_	0.49	0.19	2.53	2.33 × 10^−2^ *
*X*_1_ *X*_3_	−0.09	0.19	−0.45	6.57 × 10^−1^
*X*_1_ *X*_4_	0.04	0.19	0.21	8.39 × 10^−1^
*X*_2_ *X*_3_	0.45	0.19	2.35	3.18 × 10^−2^ *
*X*_2_ *X*_4_	0.07	0.19	0.35	7.31 × 10^−1^
*X*_3_ *X*_4_	0.93	0.19	4.84	1.83 × 10^−4^ *

*Note: X*_1_: pH, *X*_2_: temperature, *X*_3_: initial Cr(II) concentration, *X*_4_: contact time.

* Statistically significant.

In the study where adsorption of Cr(III) onto acid activated with 2 mol/L H_2_SO_4_ perlite immobilized on *Pichia kudriavzevii* JD2 yeast was examined by experimental design method; regression analysis using the significance *F* value and model *F* value showed that the model was statistically significant.

#### 3.7.4 Single Effects of Significant Parameters Affecting Cr(III) Adsorption

**FIGURE 14:**
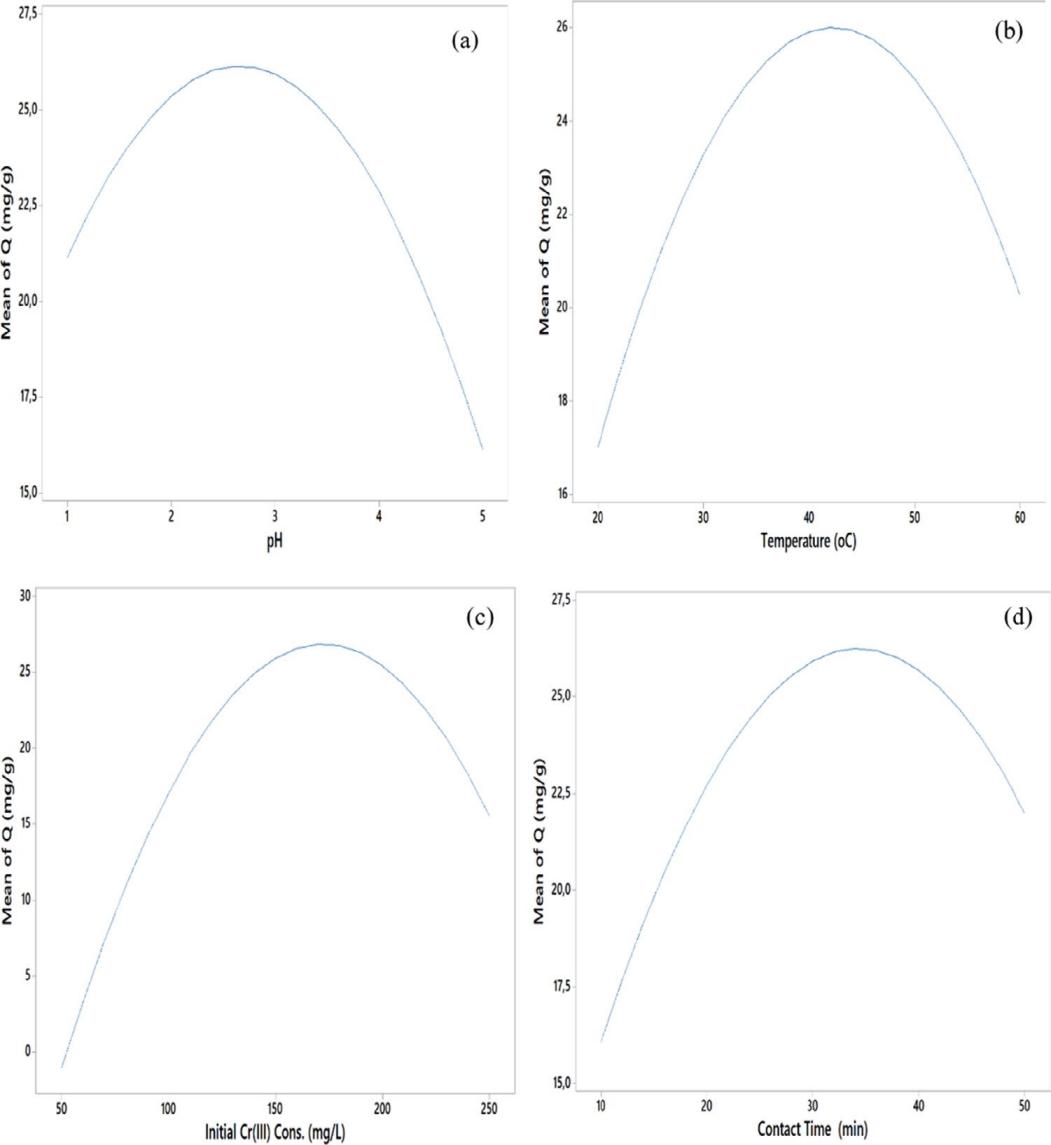
Effect of main parameters at Cr(III) adsorption onto 2 mol/L H_2_SO_4_ acid activated perlite immobilized on *Pichia kudriavzevii* JD2 yeast.

The impact of solution pH on adsorption of Cr(III) onto acid activated with 2 mol/L H_2_SO_4_ perlite immobilized on *Pichia kudriavzevii* JD2 yeast boosted in the pH range of 1.00–2.75 and decreased in the pH range of 2.75–5.0 (Figure 14a). The maximum Cr(III) adsorption was achieved at pH 2.7 conditions, with a *Q* value of 26.1 mg/g. In temperature effect, there is an increase in Cr(III) adsorption at 20°C–40°C, a decrease is observed at 40°C–60°C. An enhance in biosorption efficiency was also determined due to the formation of new active sites on the biosorbent surface with the increase in temperature. The maximum Cr(III) adsorption was found at 42.6°C conditions with a *Q* value of 27.8 mg/g (Figure 14b). Cr(III) adsorption in the range of 50–175 mg/L, a decrease is observed in the range of 175–250 mg/L. Depending on the increasing Cr(III) concentration, the increase in the amount of Cr(III) adsorbed in milligrams per gram of biosorbent is shown. The highest Cr(III) adsorption was obtained at 175 mg/L Cr(III) concentration conditions with a *Q* value of 27.7 mg/g (Figure 14c). The impact of contact time increased in the contact time at 10–35 min and decreased at 35–50 min (Figure 14d). The maximum Cr(III) adsorption *Q* value was found as 27.7 mg/g under conditions of contact time of 36 min.

#### 3.7.5 The Combined Effects of Significant Parameters Affecting Cr(III) Adsorption

**FIGURE 15:**
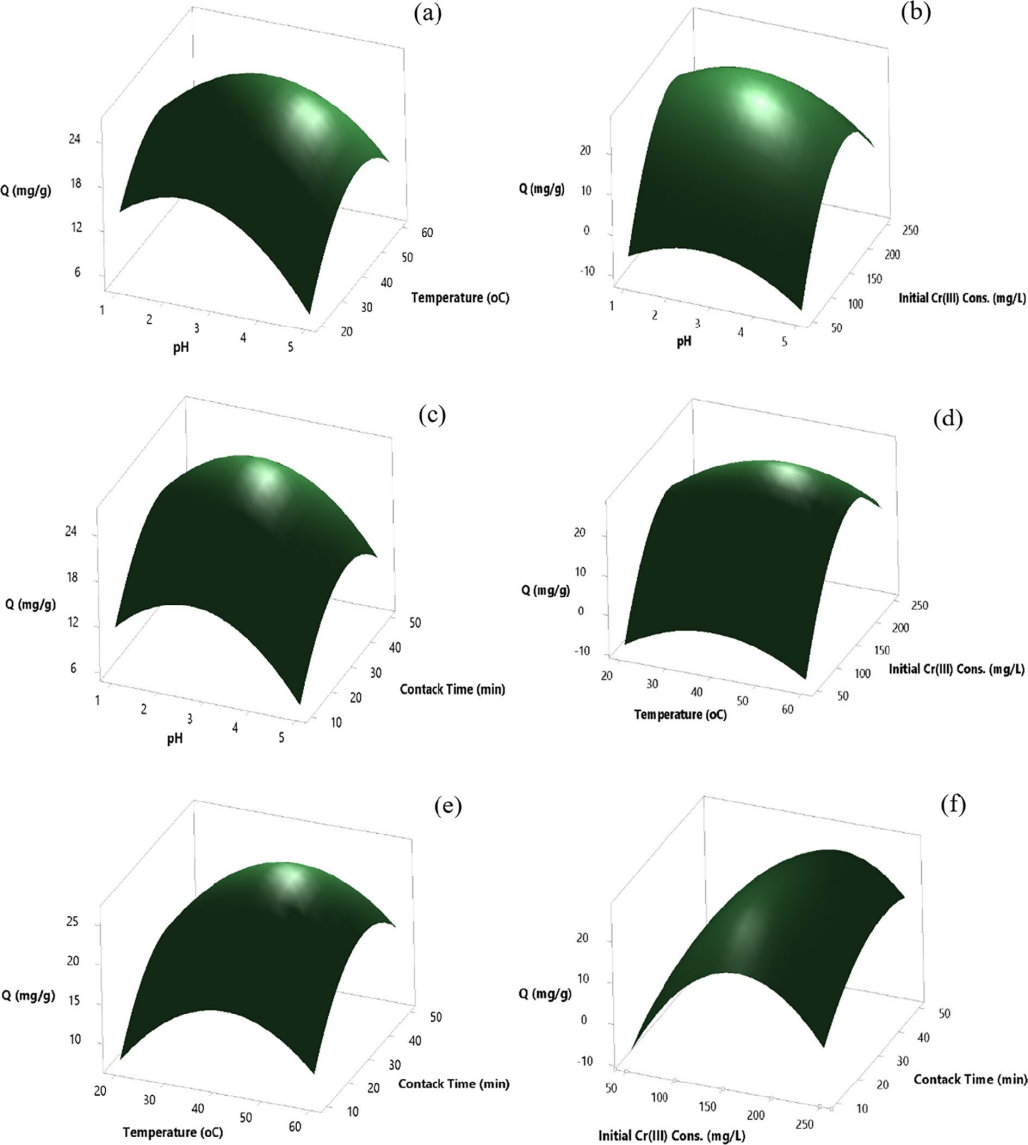
Response surface graphs: (a) pH and temperature, (b) pH and initial Cr(III) concentration, (c) pH and contact time, (d) temperature and initial Cr(III) concentration, (e) temperature and contact time, (f) initial Cr(III) concentration and contact time.

The combined effects of significant parameters affecting Cr(III) adsorption were exhibited in 3D surface plots with factors at fixed levels (Figure 15a–f). The *Q* value of the maximum Cr(III) biosorption was found as 27.7 mg/g under conditions where pH was 2.7 and temperature was 42.6°C (Figure 15a). The *Q* value of the maximum Cr(III) biosorption was discovered as 27.0 mg/g under the conditions of 43°C and 173 mg/L initial Cr(III) concentration (Figure 15d). The highest Cr(III) biosorption was obtained as 27.4 mg/g at an initial Cr(III) concentration of 175 mg/L and 36 min contact time (Figure 15f). Changes in binary interactions pH and initial Cr(III) concentration, contact time and temperature and contact time did not have statistically significant effects on biosorption (Figure 15b,c,e).

**FIGURE 16:**
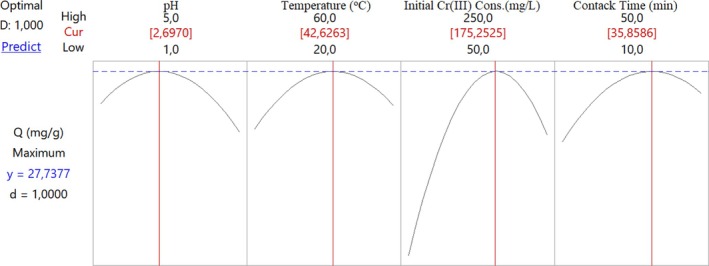
Optimum values of parameters according to CCD model with four independent variables for adsorption of Cr(III) onto acid activated with 2 mol/L H_2_SO_4_ perlite immobilized on *Pichia kudriavzevii* JD2 yeast.

*Q* values in mg of Cr(III) adsorbed per gram of adsorbent were determined under optimum conditions (pH: 2.70, temperature: 42.6°C, initial Cr(III) concentration: 175.2 mg/L, and contact time: 35.5 min). It was determined to be relatively low (27.74 mg/g). (Figure 16).

## 4 Conclusion

The novelty of this study lies in its being the first to investigate the removal of Cr(III) from the environment using *P. kudriavzevii* JD2 yeast immobilized perlite. The proposed biosorption method of Cr(III) from water samples had successfully applied. The method is sensitive, simple, accurate, speed, environment‐friendly, and low‐cost. The presence of other ions (K^+^, Mg^2+^, Ca^2+^, Cu^2+^, Cd^2+^, Mn^2+^, Pb^2+^, Ni^2+^, Zn^2+^, Fe^3+^, Co^2+^) in the water did not hinder the removal of Cr(III), demonstrating the selectivity of the process. All preconcentration procedure were accomplished within an hour, highlighting the efficiency of the method. A small amount of yeast biomass immobilized on perlite is sufficient to remove Cr(III). While the surface area of raw perlite was 7.99 m^2^/g, the surface area of the sample treated with 2 mol/L H_2_SO_4_ acid increased to 11.99 m^2^/g. Acid activation resulted in a two‐fold enhance in the surface area of perlite without affecting its structural properties. Sorption behavior of Cr(III) on 2.0 mol/L H_2_SO_4_ treated perlite and yeast immobilize perlite was conducted and main parameters influencing the experiments were investigated with CCD. Kinetic studies showed that Cr(III) adsorption followed a pseudo‐second‐order model. Thermodynamic analysis revealed that the adsorption process was endothermic. As a sustainable and environmentally friendly material, immobilized yeast perlite offers a viable option for treating wastewater contaminated with Cr(III), providing a more eco‐conscious approach to pollution control.

## Author Contributions

**Aliya Amanzhol:** investigation, writing – original draft, methodology. **Özcan Yalçınkaya:** supervision, conceptualization, funding acquisition, writing – original draft, project administration. **Berat Çinar Acar:** investigation, writing – original draft, methodology. **Zehranur Yuksekdag:** investigation, writing – original draft.

## Ethics Statement

The authors have nothing to report.

## Consent

The authors have nothing to report.

## Conflicts of Interest

The authors declare no conflicts of interest.

## Data Availability

The data that support the findings of this study are available on request from the corresponding author. The data are not publicly available due to privacy or ethical restrictions.
